# Increased scale-free and aperiodic neural activity during sensorimotor integration—a novel facet in Tourette syndrome

**DOI:** 10.1093/braincomms/fcab250

**Published:** 2021-10-27

**Authors:** Nico Adelhöfer, Theresa Paulus, Moritz Mückschel, Tobias Bäumer, Annet Bluschke, Adam Takacs, Eszter Tóth-Fáber, Zsanett Tárnok, Veit Roessner, Anne Weissbach, Alexander Münchau, Christian Beste

**Affiliations:** 1 Cognitive Neurophysiology, Department of Child and Adolescent Psychiatry, Faculty of Medicine, TU Dresden, 01069 Dresden, Germany; 2 Institute of Systems Motor Science, University of Lübeck, 23562 Lübeck, Germany; 3 Department of Neurology, University of Lübeck, 23538 Lübeck, Germany; 4 Doctoral School of Psychology, ELTE Eötvös Loránd University, 1064 Budapest, Hungary; 5 Institute of Psychology, ELTE Eötvös Loránd University, 1053 Budapest, Hungary; 6 Vadaskert Child and Adolescent Psychiatry Hospital and Outpatient Clinic, 1021 Budapest, Hungary; 7 Cognitive Psychology, Faculty of Psychology, Shandong Normal University, Qianfoshan Campus, No. 88 East Wenhua Road, Lixia District, Ji’nan, 250014, China

**Keywords:** Tourette syndrome, signal-to-noise ratio, dopamine, cognition

## Abstract

Tourette syndrome is a common neurodevelopmental disorder defined by multiple motor and phonic tics. Tics in Tourette syndrome resemble spontaneously occurring movements in healthy controls and are therefore sometimes difficult to distinguish from these. Tics may in fact be mis-interpreted as a meaningful action, i.e. a signal with social content, whereas they lack such information and could be conceived a surplus of action or ‘motor noise’. These and other considerations have led to a ‘neural noise account’ of Tourette syndrome suggesting that the processing of neural noise and adaptation of the signal-to-noise ratio during information processing is relevant for the understanding of Tourette syndrome. So far, there is no direct evidence for this. Here, we tested the ‘neural noise account’ examining 1/*f* noise, also called scale-free neural activity as well as aperiodic activity, in *n* = 74 children, adolescents and adults with Tourette syndrome and *n* = 74 healthy controls during task performance using EEG data recorded during a sensorimotor integration task. In keeping with results of a previous study in adults with Tourette syndrome, behavioural data confirmed that sensorimotor integration was also stronger in this larger Tourette syndrome cohort underscoring the relevance of perceptual-action processes in this disorder. More importantly, we show that 1/*f* noise and aperiodic activity during sensorimotor processing is increased in patients with Tourette syndrome supporting the ‘neural noise account’. This implies that asynchronous/aperiodic neural activity during sensorimotor integration is stronger in patients with Tourette syndrome compared to healthy controls, which is probably related to abnormalities of GABAergic and dopaminergic transmission in these patients. Differences in 1/*f* noise and aperiodic activity between patients with Tourette syndrome and healthy controls were driven by high-frequency oscillations and not lower-frequency activity currently discussed to be important in the pathophysiology of tics. This and the fact that Bayesian statistics showed that there is evidence for the absence of a correlation between neural noise and clinical measures of tics, suggest that increased 1/*f* noise and aperiodic activity are not directly related to tics but rather represents a novel facet of Tourette syndrome.

## Teaser

Adelhöfer et al. report that 1/*f* noise, as well as aperiodic activity during sensorimotor processing, is increased in patients with Tourette syndrome. Authors also report that increased 1/*f* noise and aperiodic activity are not directly related to tics suggesting that these findings represent a novel facet of Tourette syndrome.

## Graphical Abstract

**Graphical Abstract:**
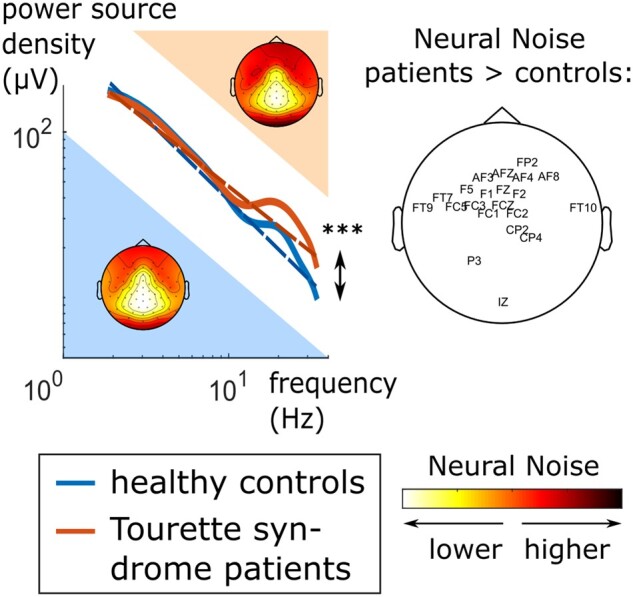

## Introduction

Tourette syndrome is a common neurodevelopmental disorder defined by multiple motor and phonic tics, often associated with preceding urges, with an onset before the age of 18 and a duration of at least 1 year.^1^ One of the key characteristics of tics in patients with Tourette syndrome is their resemblance to spontaneously occurring movements in healthy controls, so that it can be difficult to discern single tics from single spontaneous movements in healthy people.^2^^,^^3^ Thus, from the perspective of an observer, particularly if unfamiliar with tics/Tourette syndrome, tics, e.g. winking or staring, may be mis-interpreted as a socially meaningful action, i.e. a signal with social content,^4^ whereas tics lack such information. They rather represent meaningless movements (in terms of social interaction) and could therefore be considered a surplus of action or ‘motor noise’.^5^ This interpretation is supported by the observation that people with tics, particularly children, are often unaware of their tics.^6^^,^^7^ Also, adult patients with Tourette syndrome typically underestimate frequency and repertoire of their tics.^6^ Since even Tourette syndrome specialists have difficulties to discern tics from spontaneously occurring movements in healthy people,^2^^,^^3^ it is possible that a suboptimal distinction between tics as noise and other response options might be a core neurobiological problem in Tourette syndrome. Based on such clinical considerations, the ‘neural noise account of Tourette syndrome’ has recently been put forward.^8^ This account suggests that the processing of neural noise and adaptation of the signal-to-noise ratio during information processing may be central to understand the nature of Tourette syndrome.^8^ In addition to clinical observations, experimental data also suggest that altered processing of noise may indeed be evident in Tourette syndrome.^8^ For instance, reduced sensory and sensorimotor gating in Tourette syndrome^9^ is expected to lead to increased sensorimotor noise. Also, some data imply that in patients with Tourette syndrome tics are (mis-)interpreted as relevant action-related information (i.e. a relevant signal) that needs to be gated/controlled,^9^^,^^10^ suggesting that these patients have problems to discern relevant and less relevant activity patterns. In addition, findings that patients with Tourette syndrome have difficulties in action-oriented predictive processing^11^ and have less precise (i.e. more noisy) forward model estimates during action planning^10^ suggest that increased sensorimotor noise is central for the understanding of Tourette syndrome.^8^ According to the neural noise account of Tourette syndrome,^8^ increased noise levels in neural activity not only lead to spontaneous tic movements,^12^ but can also explain problems in patients with Tourette syndrome during decisions ‘when’ to carry out a voluntary action,^13^ which has repeatedly been shown.^2^^,^^14^^,^^15^ Moreover, the neural noise account may also explain patterns of concomitantly increased and diminished cognitive functions in patients with Tourette syndrome, as reviewed previously.^8^

In spite of these findings motivating the neural noise account of Tourette syndrome, there is as yet no direct evidence for the hypothesis that noise during sensorimotor processing is indeed increased in patients with Tourette syndrome and reflects a novel, important facet of this disorder.^8^ However, it has been suggested that the ‘neural noise account’ can be tested examining 1/*f* noise as an estimate for so-called ‘pink noise’,^16^ also referred to as scale-free or arrhythmic neural activity,^17^ using neurophysiological data. Importantly, the 1/*f* noise does not capture meaningless unstructured noise.^17^ Rather, it captures specific organizations relevant to information processing and brain functioning,^17–19^ which are also modulated during sensorimotor processes^20–23^ and could be measured using EEG data.^19^^,^^21^^,^^22^^,^^24^^,^^25^ Therefore, in this study, we measured 1/*f* noise in a large group of children, adolescents and adults with Tourette syndrome using EEG during an established sensorimotor integration task.^26^ We focussed on a sensorimotor integration task because multiple lines of evidence suggest that the integration of perception and action, and less so the integration of different motor processes, plays a critical role in the pathophysiology of Tourette syndrome.^5^^,^^26–32^ We hypothesize that there is stronger 1/*f* noise, i.e. scale-free activity, during sensorimotor processing in patients with Tourette syndrome than in healthy controls. More recently, the 1/*f* method has been criticized, especially with respect to its applicability in the context of event-related data,^33^ the reason being that the 1/*f* method can conflate narrow-band power with the broader-band 1/*f* component. As a consequence, by calculating 1/*f* it cannot be ruled out that possible differences between patients with Tourette syndrome and healthy controls in aspects attributable to scale-free activity when applying 1/*f* do in fact reflect changes in oscillatory power. By applying the so-called ‘fitting oscillations & one-over f’ (FOOOF) algorithm,^33^ one can control for these problems. Applying this algorithm, it has been shown that period and aperiodic oscillatory activity can reliably be distinguished.^33^ The so-called aperiodic oscillatory activity, showing a 1/*f*-like distribution,^33^ refers to arrhythmic activity and thus activity that is not specific for a particular frequency band (similar to 1/*f* activity). ‘Aperiodic activity’ has been used by the developers of the FOOOF method to refer to any activity that is non-periodic (https://fooof-tools.github.io/fooof/faq.html) as a theoretically ‘neutral’ descriptor. For the data analysis, we use both, the 1/*f* method and the FOOOF method to provide a balanced presentation of the study’s findings.

In explorative analyses, we also examined possible relations of 1/*f* noise and aperiodic activity during sensorimotor processing with clinically relevant parameters capturing tic severity. If there are substantial correlations with these parameters, this will suggest that the clinical phenotype is due to increased 1/*f* noise or aperiodic activity. However, if there is substantial evidence for the absence of such correlations, but at the same time patients with Tourette syndrome differ from healthy controls in the strength of 1/*f* noise and aperiodic activity, this would suggest that 1/*f* noise and aperiodic activity represents an additional facet of Tourette syndrome beyond tics.

## Materials and methods

### Participants

We recruited a group of *n* = 74 patients with Tourette syndrome (49 males, 25 females, mean age 19.92 ± 11.15 SD, range 8–53 years) from the specialized Tourette syndrome outpatient clinics of the Department of Paediatrics and the Department of Psychiatry and Psychotherapy at the University Medical Center Schleswig-Holstein, Campus Lübeck, Germany, from the Department of Child and Adolescent Psychiatry at the University Hospital Dresden, Germany, and the Vadaskert Child and Adolescent Psychiatry Hospital and Outpatient Clinic in Budapest, Hungary. In addition, we enrolled *n* = 74 age-related and predominantly gender-matched healthy control subjects (48 males, 26 females, mean age 19.16 ± 9.96 SD, range 8–49 years).

To assess lifetime clinical information, all participants, i.e. both patients and healthy controls, underwent a standardized clinical assessment including a clinical neuropsychiatric interview, a screening for neuropsychiatric disorders and IQ testing. To detect psychiatric comorbidities, i.e. mood disorders and obsessive compulsive disorder (OCD), we used—depending on participants’ age—the Mini International Neuropsychiatric Interview^34^ for participants over 17 years and the Mini International Neuropsychiatric Interview Kid^35^ for participants from 8 to 17 years. Attention deficit hyperactivity disorder (ADHD) symptom severity of the adult participants was evaluated using the German version of the Conners Adult ADHD Rating Scale; to rate ADHD symptoms in child and adolescent participants, the German version of the Conners third edition Rating Scales (short form) was used.^36^^,^^37^ To assess OCD symptoms, we used—depending on participants’ age—the Yale-Brown Obsessive Compulsive Scale (Y-BOCS)^38^ or the Children’s Yale-Brown Obsessive Compulsive Scale (CY-BOCS).^39^ Patients with psychosis or major depressive episode at the time of study participation were excluded. Patients with autism spectrum disorders were also excluded. All healthy controls with any indication of clinically relevant neuropsychiatric morbidity at the time of the study were excluded. The IQ was determined by using the short German version of the Hamburg–Wechsler Intelligence Scale for children for participants from 8 to 16 years^40^ or the Wechsler Adult Intelligence Scale for participants over 16 years.^41^ We only included participants with an IQ ≥ 80. Handedness was identified using the Edinburgh Handedness Inventory.^42^ A standardized video was taken of each participant, i.e. patients and healthy controls, and independently scored by two experienced examiners using the Modified Rush Videotape Rating Scale^43^ with a total tic score ranging from 0 to 20. When scores differed, a consensus was reached after discussing all relevant segments of the standardized video. In addition to a Rush consensus score, we determined motor tic frequency (tics/min). Lifetime tics and tic severity of the patients with Tourette syndrome were evaluated by the Diagnostic Confidence Index^44^ and by the Yale Global Tic Severity Scale (YGTSS),^45^ respectively. Premonitory urges were rated using the Premonitory Urge for Tic Scale.^46^

The study was performed in accordance with the Declaration of Helsinki and was approved by the local ethics committee (reference number 17–156). All patients and healthy controls (as well as the legal guardians of children and adolescent patients) provided written informed consent for study participation.

### Sensorimotor integration task

**Figure 1:**
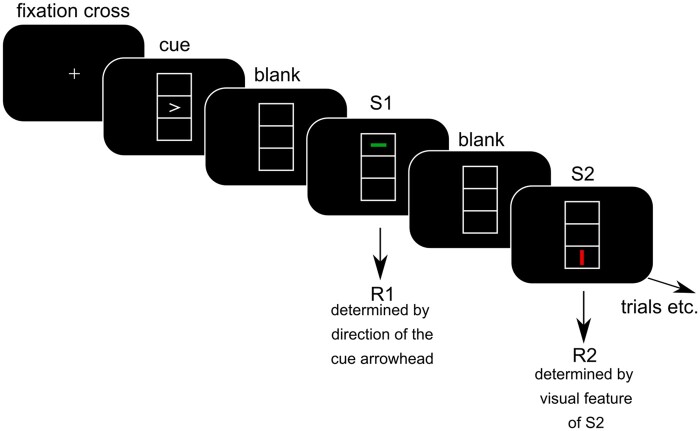
**Event file coding paradigm. Schematic illustration of the event file coding paradigm used in this study. For further details please see the main body of the manuscript**.

To examine sensorimotor integration processes in Tourette syndrome, we used a task that was already used in a previous study by our group, and which is based on the theory of event coding (TEC) framework.^47^ Briefly, this framework assumed that stimulus and response-related processes become connected with each other and thereby allow sensorimotor integration. According to TEC, this integration is achieved in so-called ‘event files’. Therefore, we used a standard event file task that was also used previously in the context of Tourette syndrome. The task is shown in Fig.  1 and described below.

Participants were seated in front of a 25″ High-Definition Multimedia Interface screen (60 cm viewing distance). On the screen (black background), a vertically aligned rectangle with a size of 6.7 cm × 2.8 cm was presented centrally. The rectangle itself was divided into three areas, each measuring 2.23 cm  × 2.8 cm. At the beginning of each trial, an arrow was presented in the middle box of the vertical rectangle for 1500 ms, pointing either to the left or to the right. This arrow acted as a cue stimulus. After the presentation of the cue stimulus, a blank screen was presented for 1000 ms, followed by stimulus S1. S1 could have different features and consisted of a vertical or horizontal line displayed in the top or bottom box with the colour red or green. In this respect, S1 was made up of three features. The S1 was presented on the screen for 500 ms and varied randomly in orientation (vertical/horizontal), colour (red/green) and position (top/bottom). After the presentation of S1, a blank screen was presented for 2000 ms. This was followed by the presentation of the S2 stimulus. S2 could share one or more features or differed in all features with S1. Thus, in the course of a trial, different overlaps in the visual features between the S1 and the S2 stimulus occurred (full feature overlap, no feature overlap or overlaps in one or two visual features). In each trial, two responses (R1 and R2) had to be made (by pressing the left or right control key on a QWERTZ computer keyboard). R1 was a response to the cue (arrowhead) in the direction of the arrow, i.e. right key when the arrowhead pointed to the right and vice versa. Crucially, participants were instructed not to respond immediately after the presentation of the cue stimulus, but only after S1 appeared. This relationship of S1 and R1 establishes an automatic association (binding) of S1 and R1, thus leading to the formation of an event file. The second response was to the S2 stimulus. Here, participants had to respond by pressing the left button when a horizontal line was presented and the right button when a vertical line was presented. Thus, in addition to the different feature overlap conditions (see above), there were two different response conditions: response repetition or response alternation. Participants were informed that there was no systematic relationship between S1 and R1 or between S1 and S2. Through this experimental variation, participants need to modify stimulus–response associations (binding). The canonical finding using this task is that whenever identical stimuli require different responses, previously established event file bindings cause problems because expectancies on stimulus–response associations are not or only partially fulfilled. This requires reconfiguration of the event file and integrated sensorimotor associations, which slows responses and increases error rates. In contrast, responding is facilitated whenever identical/similar stimuli trigger the same responses.^48–50^ In the task used, and in the statistical analysis of behavioural data, this binding is reflected by an interaction ‘feature overlap’ × ‘response’.^48–50^ This interaction reflects the degree of sensorimotor integration (event file binding).

The entire task comprised 384 trials, divided into three blocks of 128 trials each. The inter-trial interval was jittered between 1500 and 2000 ms, during which a fixation cross was displayed in the centre of the screen. Since modulations of sensorimotor integration are most strongly reflected in the differences between the conditions with zero and full feature overlap between the S1 and the S2 stimulus, we focussed the behavioural and EEG data analysis on these conditions. Since this approach is identical to previous work in Tourette syndrome,^26^ it is useful to test whether it is possible to replicate previous behavioural findings and modulations of sensorimotor processes in a larger sample.

### EEG recording and pre-processing

During the task, the EEG was recorded using 60 equidistant Ag/AgCl electrodes mounted in an elastic cap. The sampling rate was 500 Hz (reference at Fpz, ground electrode at *θ* = 58, ф = 78). All electrode impedances were kept below 10 kΩ. After an initial, manual raw data inspection to remove gross technical artefacts, a band-pass filter (2–40 Hz, slope: 48 dB/oct) was applied. This was followed by an independent component analysis (infomax algorithm) to detect and discard periodically occurring artefacts, such as horizontal and vertical eye movements, as well as pulse artefacts. Then, the EEG data were segmented (locked) to the onset of the S2 stimulus. Separate segments were built for all possible trial combinations of feature overlap and response (repetition versus alternation). The built segments had a length of 4000 ms, starting 2000 ms before the onset of the S2 stimulus. Only trials with correct responses to the cue and to the S2 stimulus were included in the data analysis. In the segmented data, epochs were discarded from further data analyses during the artefact rejection procedures, if at least one of the following criteria were met: amplitudes within this epoch higher than 200 μV and lower than −200 μV, voltage increases of 200 μV in a 200-ms interval as well as an activity below 0.5 μV in a 100-ms period. To remove the reference potential from the data, a current source density transformation was applied.^51^ Next, the data were baseline corrected to a time interval from −200 to 0 ms.

### Estimation of scale-free activity (1/*f* noise)

To calculate scale-free activity (1/*f* neural noise) in the segmented EEG data, a time window of interest from 0 (i.e. S2 stimulus onset) to 1000 ms after the stimulus presentation was selected. According to previous work,^21^^,^^22^ the power spectral density (PSD) for each frequency was computed using Welch’s method.^25^ This is because leakages can be induced due to the finite dataset during the estimation of the power of a time-limited signal. This is reduced by Welch’s method. Using the Welch method, each time signal (×) is split into seven segments (*L*) each with a duration of 250 ms with 50% overlap and windowed with a Hamming window (w). Subsequently, the discrete Fourier transform is computed, obtaining the modified periodograms ($X_{l}=\;F\{\times \;w\}$). The squared magnitude is calculated and, finally, the periodograms are averaged to gain the PSD estimate^52^:

$$\text{estimated}\;\text{PSD}=\;\frac{1}{L}\sum_{l=1}^{L}\left(|{X_{l}|}^{2}\right)$$

For every single trial, this procedure was run in Matlab applying the ‘pwelch’ Matlab function. To obtain the estimated PSD, the data were averaged separately over subjects, channels and conditions. To estimate 1/*f* neural noise represented by the slope (*β*) of the logarithm of the PSD, a linear regression with respect to the frequency (*f*) was calculated according to:

log(PSD) = β log(f) + ε,

where *ε* represents the error variable. A steeper slope of the 1/*f* noise functions indicates less noise in the neurophysiological data, while a flatter slope indicates more 1/*f* noise. The reason behind is that the slope of the 1/*f* noise function is determined by the level of neuronal population spiking activity as measured by local field potentials (LFP).^19^ This activity is also measured using the EEG signal.^53^^,^^54^ It has been shown that when a large number of spikes occur relatively simultaneously, the aggregate LFP 1/*f* slope will be more negative. When spiking is relatively asynchronous, the LFP 1/*f* slope will be flatter.^23^^,^^25^ Therefore, synchronized neuronal spiking activity is associated with reduced neuronal noise, whereas asynchronous spiking, related to increased neural noise levels, is associated with a flatter slope. We calculate the 1/*f* noise parameter using the frequency spectrum between 2 and 40 Hz. This was done because the 1/*f* metric has mainly been applied to measure broadband noise and it is known that particularly higher frequency band activity contributes to 1/*f* noise,^19^ even though there is evidence that scale-free dynamics is evident in narrow-band amplitude fluctuations.^55^^,^^56^

### Estimation of aperiodic activity

To estimate aperiodic activity, we used the FOOOF-toolbox^33^ (version 1.0.0) (https://fooof-tools.github.io/fooof/index.html). As outlined in recent work,^57^ the PSD *P*(*f*) is modelled as a combination of periodic [*G*_n_(*f*)] and aperiodic aspects [*L*(*f*)], where *f* is the frequency.

$$P\left(f\right)=L\left(f\right)+\;\sum_{n}G_{n}(f).$$

The aperiodic contribution *L*(*f*) is defined as

$$L\left(f\right)=b-\log[f^{\chi }].$$

Here, *b* reflects a constant offset and χ the aperiodic exponent, which corresponds to the slope of a line fitted to a log-log plotted power spectrum. The oscillatory contribution is modelled as Gaussian peak:

$$G_{n}\left(f\right)=\;a_{n}\exp[-\;\frac{{\left(f-\;\mu_{n}\right)}^{2}}{{2\sigma }_{n}^{2}}],$$

with *a_n_* as the amplitude, μ_*n*_ as the centre frequency and *σ_n_* as the bandwidth of each component (the number of oscillatory components is determined from the data).^57^ Precisely, the following steps were taken to calculate the aperiodic measure using the FOOOF toolbox: we used data segments from stimulus onset until 1000 ms. In this time window, the PSD was calculated as described above for the 1/*f* analysis. The aperiodic exponent was fitted on the single-subject’s average of data from the different experimental conditions. All exponents from model fits satisfied a minimum *R*^2^ value of 0.95.

### Statistical analyses

Behavioural data (i.e. reaction times and error rates) and the 1/*f* noise parameter were analysed using mixed effects ANOVAs. The factor ‘group’ (healthy control versus Tourette patients) was used as a between-subject factor. The factors ‘feature overlap’ and ‘response’ were used as within-subject factors. Greenhouse–Geisser correction was applied for tests, if necessary. *Post-hoc* tests were Bonferroni-corrected, if necessary. Regression and correlation analyses, including Bayesian statistics, were calculated between clinical parameters and the 1/*f* noise parameter. Analyses were conducted using SPSS and JASP software packages.

### Data availability

Anonymized data can be shared by request from any qualified investigator. Data will be available for 10 years.

## Results

### Clinical data of the participants

Based on the interview assessment, 14 patients with Tourette syndrome had depression in the past. Four patients had a depressive episode, and two patients had anxiety disorder at the time of testing. In addition, 9 patients with Tourette syndrome had ADHD and 10 patients had OCD. Clinical characteristics of patients with Tourette syndrome are shown in Table  1. At the time of study participation, nine patients were taking medication including aripiprazole (*n* = 4), tiapride (*n* = 3), methylphenidate (*n* = 3) and atomoxetine (*n* = 1). One patient with Tourette syndrome consumed cannabis on a regular basis.

**Table 1:** Clinical characteristics of patients with Tourette syndrome

Clinical scale	Minimum	Maximum	Mean	SD
Disease duration (years)	0.25	43	11.5	10.5
YGTSS total (0–100)	4	91	34.7	19.0
YGTSS tics (0–50)	4	91	20.4	12.4
Rush total score (0–20)	1	19	10.6	4.0
Rush, tics per minute	2	111	38.6	4.6
DCI (0–100)	16	100	51.8	19.9
CY-BOCS/Y-BOCS (0–40)	0	26	4.6	6.6
PUTS (0–36)	1	19	18.9	9.8
ADHD IA, T-score (adults)	37	90	57.3	13.3
ADHD HI, T-Score (adults)	36	84	53.4	13.6
ADHD IA, T-Score (children, adolescents)	34	73	54.1	9.8
ADHD HI, T-Score (children, adolescents)	40	73	56.1	8.1

ADHD = attention deficit hyperactivity disorder; CY-BOCS = Children's Yale-Brown Obsessive Compulsive Scale; DCI = Diagnostic Confidence Index; HI = hyperactivity/impulsivity; IA = inattention; PUTS = Premonitory Urge for Tics Scale; Rush = Rush Video-Based Tic Rating-Scale; Y-BOCS = Yale-Brown Obsessive Compulsive Scale; YGTSS = Yale Global Tic Severity Scale.

According to the interview assessment, seven healthy controls had psychiatric comorbidities. Four had depression in the past, one had panic attacks and depression in the past, one had panic attacks in the past and one had a hypomanic episode in the past. Importantly, none of the healthy controls had any clinically relevant psychiatric symptomatology during their study participation.

Mean IQ was 104.7 (±12.5) in patients with Tourette syndrome and 111.1 (±10.4) in healthy controls. A total of 13 patients and 7 healthy control subjects were left-handed.

### Behavioural data

**Figure 2:**
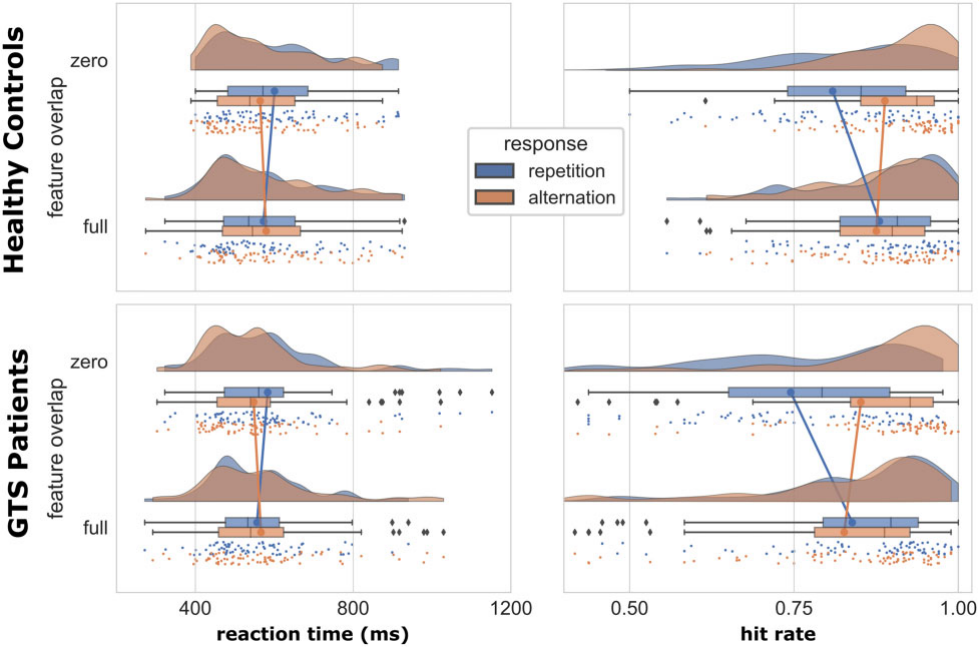
**Behavioural data in patients with Tourette syndrome and healthy controls.** Full depiction of the behavioural data (mean response times and hit rates) in patients with Tourette syndrome and healthy controls as a function of task conditions (feature overlap and response). Each plot shows the individual data as a scatterplot (*y* axis jitter added for discernibility) in addition to boxplot information and a density-based distribution. Illustrations were accomplished using the Raincloud plot toolbox.^83^ Outliers are indicated by diamonds. For the hit rates, the data underlying the significant interaction ‘group’×‘feature overlap’×‘response’ [*F*(1,146) = 4.227; *P* = 0.042; *η*_p_2 = 0.028] are shown. This interaction was not significant for the reaction time data shown [*F*(1,146) ≤ 1.286; *P* ≥ 0.259].

An overview of the behavioural data is shown in Fig.  2.

For the reaction times, the ANOVA revealed a main effect ‘response’ [*F*(1,146) = 9.388; *P* = 0.003; *η*_p_2 = 0.060]; participants responded faster during trials with repeated (564.44 ± 11.1 ms) compared to alternating responses (578.23 ± 11.3 ms). An interaction effect between task conditions (i.e. ‘feature overlap’ × ‘response’) could be shown as well [*F*(1,146) = 46.137; *P* < 0.001; *η*_p_2 = 0.240]. More precisely, within response repetition trials, reaction times were significantly longer in zero feature overlap trials (592.0 ± 12 ms) compared to full feature overlap trials (564.5 ± 10 ms) [*t*(147) = 4.89; *P* < 0.001; *d* = 0.195]. In alternating response trials, however, this pattern was reversed (zero feature overlap: 556.0 ± 10 ms; full feature overlap: 572.9 ± 12 ms) [*t*(147) = 2.93; *P* = 0.004; *d* = 0.122]. This pattern of results reflects known effects of stimulus-response binding, i.e. sensorimotor integration.^26^^,^^58^^,^^59^ No other factor between or within subjects, or interactions between them, resulted in statistical significance [all other *F*(1,146) ≤ 1.286; *P* ≥ 0.259].

The ANOVA on hit rates revealed two main effects that matched the experimental conditions. First, full feature overlap resulted in higher hit rates (0.85 ± 0.02) compared to zero overlap trials (0.82 ± 0.03) [*F*(1,146) = 7.825; *P* = 0.006; *η*_p_2 = 0.051]. The other main effect [‘response’; *F*(1,146) = 20.087; *P* < 0.001; *η*_p_2 = 0.121] shows more accurate performance in alternating (0.86 ± 0.02) compared to repeating response trials (0.82 ± 0.02). The interaction ‘group’ × ‘feature overlap’ × ‘response’ turned out to be statistically significant as well [*F*(1,146) = 4.227; *P* = 0.042; *η*_p_2 = 0.028]. As mentioned, an interaction ‘feature overlap’ × ‘response’ shows that stimulus and motor response processes become integrated and bound into an event file.^26^^,^^58^^,^^59^ The interaction ‘group’ × ‘feature overlap’ × ‘response’ thus shows that the event file binding or the degree of sensorimotor integration differs between patients with Tourette syndrome and healthy controls, which was also previously shown in a smaller adult sample of patients with Tourette syndrome.^26^ Kleimaker et al.^26^ showed that the sensorimotor integration (event file binding) was stronger in patients with Tourette syndrome than healthy controls. The same is evident in this study in a more extended sample of patients because the effect size (i.e. *η*_p_^2^) of the interaction ‘feature overlap’ × ‘response’ was larger in the Tourette syndrome group [*F*(1,73) = 87.301; *P* < 0.001; *η*_p_2 = 0.545] than in the healthy control group [*F*(1,73) = 56.536; *P* < 0.001; *η*_p_2 = 0.436]. No other main or interaction effects were significant [all other *F*(1,146) ≤ 0.275; *P* ≥ 0.601].

### 1/*f* data

**Figure 3:**
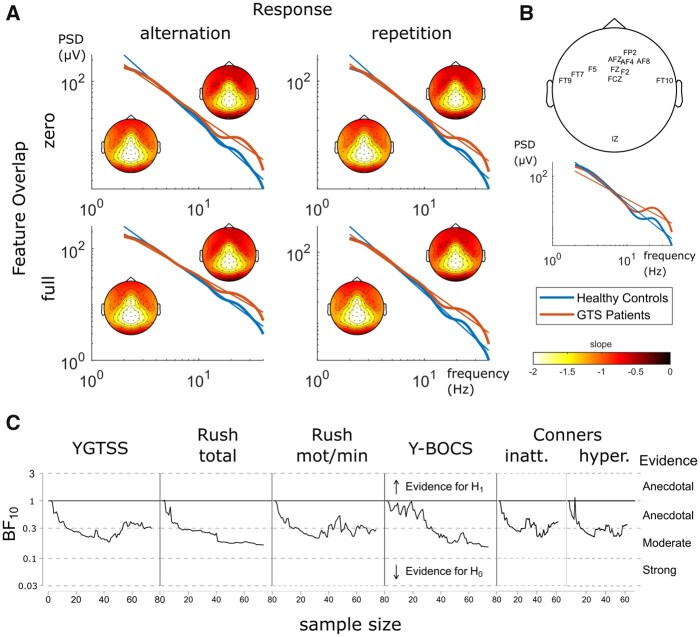
**Results of 1/*f* analyses.** (**A**) Log–log-transformed PSD data of SR task conditions averaged across electrodes in the different groups (Tourette patients and healthy controls). Neural noise is defined as the respective log_10_-transformed PSD slope parameter with more positive values indicating higher neural noise. Scalp topographies are illustrated for each group and condition (Tourette patients: upper right corner; healthy control group: lower left corner). (**B**) Top: electrode sites with significant differences of neural noise between groups. Significant values underwent false discovery rate correction using the Benjamini–Hochberg method^84^; (*q*<0.05). Bottom: PSD plots averaged over this electrode set and task conditions. (**C**) Bayes factors obtained for the correlations between neural noise and clinical scales as a function of sample size. All Bayes factors (BF_10_<1; detailed values ranged between 0.15 and 0.67 depending on clinical parameter examined as shown in Table  2) indicate moderate evidence that no relationship between neural noise and the respective clinical scale exists in this sample. Note that for clarity Bayes factors in this plot are based on a bivariate model, which is why they diverge from values in Table  2. GTS = Gilles de la Tourette syndrome; Rush total = Rush video-based rating, total score; Rush mot/min = Rush video-based rating, motor tics per minute; Y-BOCS = Yale-Brown Obsessive Compulsive Scale; YGTSS = Yale Global Tic Severity Scale (total score).

An overview of the results of the 1/*f* analysis is depicted in Fig.  3.

Figure  3A shows the log–log plots of the PSD for patients with Tourette syndrome (red) and healthy controls (blue) for the different experimental conditions as well as linear functions fitted to the data. The slope of these linear functions provides an estimate of the 1/*f* activity. As mentioned before (cf. Materials and methods section), a steeper slope of the 1/*f* noise functions (more negative parameter) indicates less noise in the neurophysiological data, while a flatter slope (less negative parameter) indicates more 1/*f* noise. As can be seen in Fig.  3A, the slope of the 1/*f* functions appears to be flatter in patients with Tourette syndrome than healthy controls. This is also reflected in the statistical analysis. In the analysis of the mean 1/*f* parameter of all electrodes, a main effect of group was evident [*F*(1,146) = 6.691; *P* = 0.011; *η*_p_2 = 0.044]. Noise parameters indicate higher neural noise in patients with Tourette syndrome (−1.25 ± 0.10) compared to the healthy control group (−1.44 ± 0.10). Furthermore, we found an interaction effect between experimental conditions (i.e. response × feature overlap) [*F*(1,146) = 6.554; *P* = 0.011; *η*_p_2 = 0.043]. This can be pinpointed to lower noise in alternating response trials (−1.36 ± 0.04) compared to repeating response trials (−1.33 ± 0.04) when there was no feature overlap [*t*(147) = 3.15; *P* = 0.002; *d* = 0.059]. However, no such difference was found in full feature overlap trials [*t*(147) = 0.19; *P* = 0.851]. No other main or interaction effects of the group or experiment factors could be found [all other *F*(1,146) ≤ 3.831; *P* ≥ 0.052]. However, although the parameter representing the mean 1/*f* noise revealed a difference between the patients with Tourette syndrome and the healthy controls it is possible that this group difference is particularly strong at a subset of electrodes. To test this, the 1/*f* parameter was compared between groups for each electrode separately using false discovery rate correction to correct for multiple comparisons. Results are shown in Fig.  3B. It turns out that neural noise measured selectively at frontal sites differed between groups. When only using the mean of the 1/*f* noise parameter from these electrodes in the ANOVA, the same pattern as observed for an analysis using the entire set of electrodes was obtained. A significant difference between groups was observed [*F*(1,146) = 11.248; *P* = 0.001; *η*_p_2 = 0.072] with higher neural noise levels in patients with Tourette syndrome (−1.05 ± 0.11) compared to the healthy control group (−1.30 ± 0.11). However, since the effect size of this group difference was larger compared to the analysis in which the 1/*f* noise parameter was based on all electrodes, this corroborates that particularly frontal electrode sites capture differences in 1/*f* noise or scale-free activity in Tourette syndrome. Also, the interaction between task conditions turned out to be significant [*F*(1,146) = 5.4; *P* = 0.022; *η*_p_2 = 0.036]. The pattern of the interaction is similar to that across all electrodes: lower neural noise was observed in alternating response trials (−1.19 ± 0.04) compared to repeating response trials (−1.16 ± 0.04), but only in the zero feature overlap [*t*(147) = 2.87; *P* = 0.005; *d* = 0.063], not the full overlap condition [*t*(147) = 0.35; *P* = 0.724]. Again, no other main or interactive effects, including the group factor, were evident [all other *F*(1,146) ≤ 3.441; *P* ≥ 0.066].

### Aperiodic exponent data

**Figure 4:**
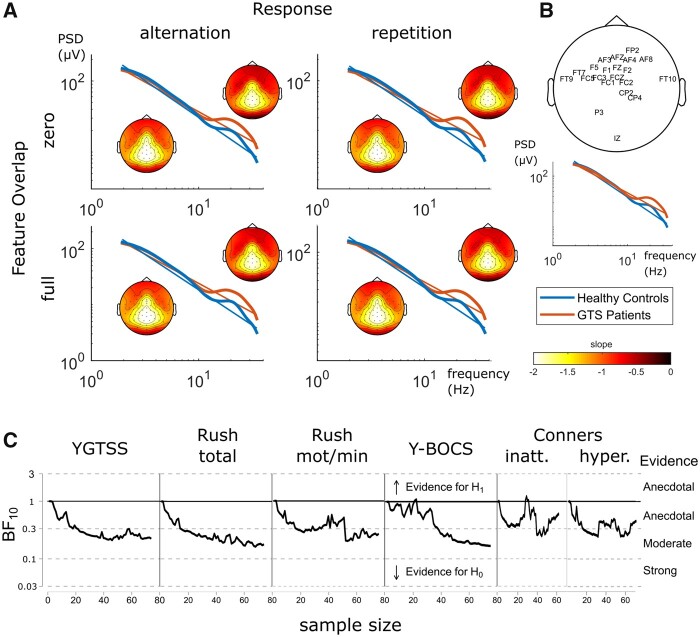
**Results of the FOOOF analyses.** (**A**) Log–log-transformed PSD data of SR task conditions averaged across electrodes in the different groups (Tourette patients and healthy controls). Neural noise is defined as the respective log_10_-transformed PSD slope parameter with more positive values indicating higher neural noise. Scalp topographies are illustrated for each group and condition (Tourette patients: upper right corner; healthy control group: lower left corner). (**B**) Top: electrode sites with significant differences of neural noise between groups. Significant values underwent false discovery rate correction using the Benjamini–Hochberg method^84^; (*q*<0.05). Bottom: PSD plots averaged over this electrode set and task conditions. (**C**) Bayes Factors obtained for the correlations between neural noise and clinical scales as a function of sample size. All Bayes factors (BF_10_<1; detailed values ranged between 0.20 and 0.86 depending on clinical parameter examined as shown in Table  2) indicate moderate evidence that no relationship between neural noise and the respective clinical scale exists in this sample. Note that for clarity Bayes factors in this plot are based on a bivariate model, which is why they diverge from values in Table  2. GTS = Gilles de la Tourette syndrome; Rush total = Rush video-based rating, total score; Rush mot/min = Rush video-based rating, motor tics per minute; Y-BOCS = Yale-Brown Obsessive Compulsive Scale; YGTSS = Yale Global Tic Severity Scale (total score).

An overview of the results of the FOOOF analysis^33^ is depicted in Fig.  4.

In the analysis of the mean aperiodic exponent of all electrodes, a main effect of group was evident [*F*(1,146) = 7.145; *P* = 0.008; *η*_p_2 = 0.047]. The aperiodic exponent, which provides the slope of a line fitted to a log–log plotted power spectrum (i.e. similar to the 1/*f* procedure) was smaller for the patients with Tourette syndrome (1.08 ± 0.05) than the healthy control group (1.27 ± 0.05), thus indicating more aperiodic activity. No other main or interaction effects of the group or experimental factors was evident [all other *F*(1,146) ≤ 3.39; *P* ≥ 0.068]. However, as with the analysis of the 1/*f* parameter, the group difference in the aperiodic exponent seemed particularly strong at a subset of electrodes. This is supported by the cluster-based permutation tests. The results are shown in Fig.  4B. When only using the mean of the aperiodic exponent from these electrodes in the ANOVA, the same pattern of results was still obtained. That is, there was a main effect of group [*F*(1,246) = 11.957; *P* < 0.001; *η*_p_2 = 0.076], showing that the aperiodic exponent was smaller for the patients with Tourette syndrome (1.03 ± 0.05) than the healthy control group (1.28 ± 0.05). No other main or interaction effects were significant [all *F*(1,146) ≤ 3.72; *P* ≥ 0.056]. Therefore, the data pattern obtained for the aperiodic exponent was comparable to the data pattern from the 1/*f* analysis.

### Correlations of the 1/*f* parameter with clinical data

In a last step, we investigated how far the 1/*f* parameter and the aperiodic exponent were correlated with clinical measures of Tourette syndrome. Since the difference between patients with Tourette syndrome and healthy controls was most prominent over frontal electrode sites, we used the mean 1/*f* parameter and the aperiodic exponent from these sites for the correlation analysis with clinical symptoms. The results are summarized in Table  2.

**Table 2:** Clinical correlations using different noise parameters (1/f noise and the aperiodic exponent over subset of electrodes)

Clinical scale	**Noise** **parameter**	Pearson *r*		**Significance (*P*)** ^a^		**Bayes factor** ^a^
		Age	**Noise** **parameter**	Age	Noise parameter	
YGTSS	1/f	0.114	−0.152	0.0215	<0.001	0.035
Rush (total score)		0.194	−0.148	0.001	<0.001	0.067
Rush (tics per minute)		0.038	−0.069	0.564	0.141	0.016
CY-BOCS/Y-BOCS		0.045	−0.050	0.300	0.061	0.015
ADHD IA, T-score^b^		0.128	−0.185	0.001	<0.001	0.050
ADHD HI, T-score^b^		−0.141	−0.174	0.017	<0.001	0.139
YGTSS	Aperiodic component	0.114	−0.103	0.041	<0.001	0.034
Rush (total score)		0.194	−0.081	0.001	<0.001	0.086
Rush (tics per minute)		0.038	−0.137	0.039	<0.001	0.047
CY-BOCS/Y-BOCS		0.045	−0.022	0.189	0.261	0.020
ADHD IA, T-score^b^		0.128	−0.214	0.003	<0.001	0.070
ADHD HI, T-score^b^		−0.141	−0.190	0.050	<0.001	0.179

a Calculation based on regression models including age and neural noise as independent variables.

b Subscale based on Conners Rating Scale for ADHD.

ADHD = attention deficit hyperactivity disorder; CY-BOCS = Children's Yale-Brown Obsessive Compulsive Scale; HI = hyperactivity/impulsivity; IA = inattention; Rush = Rush Video-Based Tic Rating-Scale; Y-BOCS = Yale-Brown Obsessive Compulsive Scale; YGTSS = Total score of Yale Global Tic Severity Scale.

Using Pearson correlations, and for both the 1/*f* parameter and the aperiodic exponent higher Tourette syndrome symptom scores (i.e. YGTSS score and Rush total score) were correlated with higher neural noise levels. Furthermore, ADHD sub-scores of inattentiveness and hyperactivity from the Conners rating scale were both significantly correlated with elevated neural noise. No significant correlations were obtained using the Y-BOCS/CY-BOCS score. For the significant correlations obtained for the 1/*f* parameter and the aperiodic exponent, the correlation coefficients were very small and did not exceed *r* = 0.2 (i.e. 4% explained variance in the data). Therefore, these correlations do not appear to be meaningful. This is corroborated using a Bayesian approach for the correlation analyses. The Bayes factors revealed strong evidence for the null hypothesis for almost all measures (the exception being the hyperactivity subscale with moderate evidence for the null hypothesis) (see also Figs  3C and 4C). It is therefore likely that statistical significance, under the frequentist approach, is affected more by the relatively large sample size rather than a meaningful effect. Therefore, the data suggest that the 1/*f* noise and the aperiodic exponent during sensorimotor integration do not relate to clinical symptom severity.

## Discussion

In this study, we tested the ‘neural noise account of Tourette syndrome’,^8^ according to which noise is increased in patients with Tourette syndrome and reflects a novel, important facet of this disorder. Given the evidence that the integration of perception and action rather than the integration of different motor processes is relevant for the understanding of Tourette syndrome,^5^^,^^26–32^ we measured 1/*f* noise, also referred to as scale-free neural activity, during an established sensorimotor integration task. In keeping with results of a previous study in adults,^26^ the pattern of results of behavioural data showed that sensorimotor integration, i.e. event file binding, was also stronger in this larger cohort of adolescents and adults with Tourette syndrome than healthy controls in this study. This underscores the relevance of considering perceptual-action processes in the pathophysiology of Tourette syndrome.

Importantly, the main finding of this study is that 1/*f* noise during sensorimotor processing is indeed increased in patients with Tourette syndrome compared to healthy controls, as hypothesized in the ‘neural noise account of Tourette syndrome’.^8^ In the data, increased 1/*f* noise in Tourette syndrome was reflected by a flatter slope of the 1/*f* function fitted to the PSD of the EEG data (Fig.  3). It is assumed that the slope of the 1/*f* noise function is predominantly determined by the level of spiking activity of neuronal populations as measured by LFPs.^19^ Synchronized neuronal spiking activity is associated with reduced neuronal noise leading to a steeper slope of the 1/*f* noise function, whereas asynchronous spiking is related to increased neural noise levels and thus reflected by a flatter slope of this function.^23^^,^^25^ Thus, our data suggest that asynchronous neural activity during sensorimotor integration is stronger in Tourette syndrome compared to healthy controls. It has been shown that particularly higher frequency activity, i.e. beta and gamma band activity, contributes to 1/*f* noise. This was the case also in this study. Corroborating the findings of the 1/*f* analysis, also performing an analysis, in which the aperiodic neural activity was calculated,^33^ revealed robust differences between groups. Thus, there is converging evidence from the application of two analysis methods. The increased 1/*f* noise predominantly in higher frequency bands in Tourette syndrome can well be explained by neurobiological abnormalities in Tourette syndrome. In this regard, it has to be noted, as outlined above, that the slope of the 1/*f* noise function is closely related to neuronal population spiking activity^19^ that is modulated by a number of factors including the activity of the gamma aminobutyric acid (GABA) system,^60^ which is of particular importance in Tourette syndrome. Neuropathological data documented a reduced number and altered distribution of inhibitory GABAergic parvalbumin-expressing and tonically active cholinergic interneurons, predominantly in the sensorimotor and associative areas of the striatum,^61^^,^^62^ which likely causes imbalances in neural activity including asynchronous spiking known to increase noise levels. Also, GABA-edited MR-spectroscopy has shown abnormalities in related to GABAergic system.^63–66^ Interestingly, aperiodic activity has been related to the net effect of excitatory (α-amino-3-hydroxy-5-methyl-4-isoxazolepropionic acid; AMPA) and inhibitory (GABA) currents with the aperiodic exponent being smaller when the inhibitory effect is smaller than the excitatory effect.^33^^,^^67^ Since the aperiodic exponent was smaller in the patients with Tourette syndrome, the analysis of aperiodic activity suggests that particularly the GABA-related dynamics in Tourette syndrome is important to consider. Yet, it cannot be ruled out that alterations of the dopaminergic system in Tourette syndrome are also likely relevant,^68^^,^^69^ particularly because it has recently been shown that increases in dopaminergic transmission can decrease 1/*f* neural noise.^21^ Against the background that patients with Tourette syndrome are probably in a hyper-dopaminergic state^68^ and the fact that anti-dopaminergic medication is the mainstay of treatment in Tourette syndrome,^68^ one might expect noise to be reduced rather than increases in Tourette syndrome. However, due to the inverted U-shape curve of dopamine functioning,^70^ noise levels can be high in a hyper-dopaminergic state.

Interestingly, differences in 1/*f* noise and aperiodic activity between patients with Tourette syndrome and healthy controls were driven by high-frequency oscillations. This is important because, at present, particularly theta oscillations seem to be relevant for processes related to tic generation. For instance, during deep brain stimulation in Tourette syndrome, theta oscillations in LFPs have emerged as characteristic in these patients both in the internal segment of the globus pallidus^71–73^ and thalamic nuclei.^74–78^ Also, synchronized oscillations in the theta range have been recorded across deep brain stimulation targets^77^ and were functionally coupled between the pallidum and the thalamus.^79^ Importantly, it was also suggested that longer theta bursts may be related to tics.^79^^,^^80^ Therefore, the obtained findings in 1/*f* noise and aperiodic activity suggest that increased noise in Tourette syndrome is not directly or solely related to processes leading to tics but rather represents a more principle abnormality in these patients. This is corroborated by the results of the correlation analysis using tics-related disease parameters. Although Pearson correlation analyses suggested that 1/*f* noise and aperiodic activity is related to clinical parameters of tic severity, i.e. the YGTSS and the Rush video score, the degree of explained variance was very small and Bayesian statistics showed that there is no substantial evidence for such a relation but in fact some evidence for the absence of such correlations. This suggests that observed modulations of 1/*f* or aperiodic activity are not directly related to tics but rather represents a novel facet of Tourette syndrome. Unlike the behavioural data, no group-dependent modulations in the strength of event file binding (sensorimotor integration effects) were reflected by the 1/*f* data. The current results of 1/*f* or aperiodic activity, possibly driven my GABA-related dynamics as discussed above, do thus reflect processes unrelated to the perception-action integration (or event file) account of Tourette syndrome.^5^^,^^28^ Interestingly, GABA-related dynamics play only a minor role in perception-action integration.^81^ This may explain why there was no correlation between 1/*f* or aperiodic activity and perception-action integration and why binding effects were not evident for the 1/*f* or aperiodic activity parameters. Moreover, event file coding seems to be mainly a function of lower-frequency (theta) band activity^59^ whereas differences in 1/*f* activity between groups were present in higher frequency bands (see Fig.  3) and aperiodic activity represents broadband dynamics. Considering the perception-action integration (or event file) account of Tourette syndrome,^5^^,^^28^ it has been argued that this account is not mutually exclusive to a social decision-making account^4^ of dysfunctions in Tourette syndrome.^82^ Future studies shall investigate whether the identified novel facet of Tourette syndrome is relevant for the social decision-making account^4^ of dysfunctions in Tourette syndrome.^82^

A possible limitation of this study relates to the specificity of our findings. Tourette syndrome is a classical spectrum disorder with ADHD and OCD being common comorbidities, so that it is conceivable that ADHD or OCD traits contributed to the main result of increased neural noise in Tourette syndrome in this study. Because of a limited number of patients with clinically relevant ADHD or OCD, reliable subgroup analyses could not be performed. Overall severity of comorbid ADHD and OCD was low in the sample we investigated. Also, Y-BOCS/CY-BOCS scores did not correlate with neural noise. In addition, on the basis of Bayes statistics, there was no evidence for a relation between ADHD severity and neural noise levels. Therefore, we consider it unlikely that traits of OCD or ADHD considerably influenced the results of this study.

To summarize, this study shows that task-related 1/*f* noise and aperiodic activity in higher frequency bands is increased during sensorimotor processing in patients with Tourette syndrome compared to healthy controls. Given the lack of evidence that scale-free and aperiodic activity as shown here are related to processes of tic generation, it is plausible to assume that such activity represents a tic-independent but disease-relevant information processing mode in Tourette syndrome. The data suggest that 1/*f* noise and aperiodic activity during sensorimotor integration reflects a novel facet of Tourette syndrome.

## Abbreviations

ADHD =: attention deficit hyperactivity disorder
CY-BOCS =: Children’s Yale-Brown Obsessive Compulsive Scale
DCI =: Diagnostic Confidence Index
FOOOF =: fitting oscillations & one-over f
LFP =: local field potential
OCD =: obsessive compulsive disorder
PFC =: prefrontal cortex
PSD =: power spectral density
PUTS =: Premonitory Urge for Tic Scale
TEC =: theory of event coding
Y-BOCS =: Yale-Brown Obsessive Compulsive Scale
YGTSS =: Yale Global Tic Severity Scale
